# Acute abdominal pain caused by hematometra in an adolescent female: a case report

**DOI:** 10.1186/s13256-016-1154-6

**Published:** 2016-12-20

**Authors:** Benjamin Chou, Jacqueline Ann Bohn, Robert Mairs

**Affiliations:** 1 Pacific Northwest University of Health Sciences, Yakima, Washington USA; 2 University of Utah, Salt Lake City, Utah USA

**Keywords:** Hematometra, Abdominal pain, Depot medroxyprogesterone acetate, Ultrasound, Adolescent, Case report

## Abstract

**Background:**

Hematometra is a pathologic collection of blood in the uterus. It is a rare condition that is most commonly associated with congenital anomalies or prior surgical procedures causing an obstruction of the genitourinary outflow tract. We present an unusual case of hematometra in a healthy and active adolescent female with no prior risk factors. This is a rare and important case report due to the complexity of diagnosis when a young female presents with an acute abdomen. In addition, for a patient who presents with no prior risk factors for hematometra, such as in our patient, the diagnosis and workup may become overly complicated, adding strain to patient care and health care cost. To the best of our knowledge and based on an extensive literature search, there has not been a reported case of hematometra in an adolescent female without any aforementioned risk factors.

**Case presentation:**

Our patient is a healthy 18 year-old white woman with no significant prior medical or surgical history. Her only medication was depot medroxyprogesterone acetate use for contraception. She presented to a local emergency department with acute abdominal pain, accompanied by emesis and nausea. Workup with ultrasonography showed uterine distention most likely caused by hematometra, although no obvious cause was noted. She was treated with dilation and curettage; she was also advised to discontinue depot medroxyprogesterone acetate use. She was symptom free without recurrence of hematometra at 6-month follow-up.

**Conclusions:**

Due to the high prevalence of abdominal pain, this case report has a wide breadth of implications for health care providers ranging from general family practitioners to emergency room physicians and obstetricians/gynecologists. This case report provides potential future advancement in management and differential diagnosis in adolescent females presenting with acute abdominal pain. In addition, the use of depot medroxyprogesterone acetate contributing to or causing hematometra cannot be ruled out in our patient and warrants further investigation.

## Background

Acute abdominal pain is one of the most common causes of emergency department (ED) visits in the USA [1]. The differential diagnosis for an acute abdomen in an adolescent female can range from appendicitis to ovarian cyst rupture to ectopic pregnancy, to name a few. A rare cause of acute abdomen in this population is hematometra, which is an inappropriate collection of blood in the uterus. This rare medical condition can lead to lower abdomen distention, pain, and discomfort [2]. Hematometra can be caused by congenital defects such as transverse uterine septum, cervical atresia, or stenosis [3]. It can also be acquired secondarily through cervical and uterine procedures such as loop electrical excision procedure (LEEP), uterine ablation, and cone biopsy of the cervix [3].

We present a case that is unusual due to the patient being an adolescent female with no congenital defects or prior history of the aforementioned risk factors associated with hematometra.

## Case presentation

An 18-year-old, gravida 0, white woman presented to an ED with acute lower abdominal pain. The pain began gradually during her strenuous sporting practice hours earlier, limiting her ability to participate. She localized the pain to her lower abdomen, describing it both as similar to menstrual cramps and reminiscent of her appendicitis 3 years ago. Hours later, she experienced nausea and emesis. She found pain relief when crouching, exacerbation during emesis and micturition, and no relief with nonsteroidal anti-inflammatory drugs.

She was previously healthy and physically active, with no surgical history besides an uncomplicated appendectomy. Menarche was at 12-years old and her menstrual cycles were regular, not painful, and occurred every 60 days. She was previously sexually active, with no history of sexually transmitted infections. Her last menstrual period was 7 months prior to her current symptoms. She had a 3-year history of depot medroxyprogesterone acetate (DMPA) use for contraception. Her family and social history were otherwise non-contributory.

On examination, both her lower abdominal quadrants were severely tender to light and deep palpation, without rigidity, rebound tenderness, or palpable masses. A pelvic examination was not performed. She was afebrile; her vital signs were age appropriate and stable. A complete blood count showed an elevated white blood cell count at 16.4×10^3^/μL (reference range 3.8 to 11.0) with no left shift on automated differential. Her pregnancy test was negative. The ED provider listed menstrual cramps as the main differential diagnosis at the time and she was sent for pelvic ultrasonography.

**Fig. 1:**
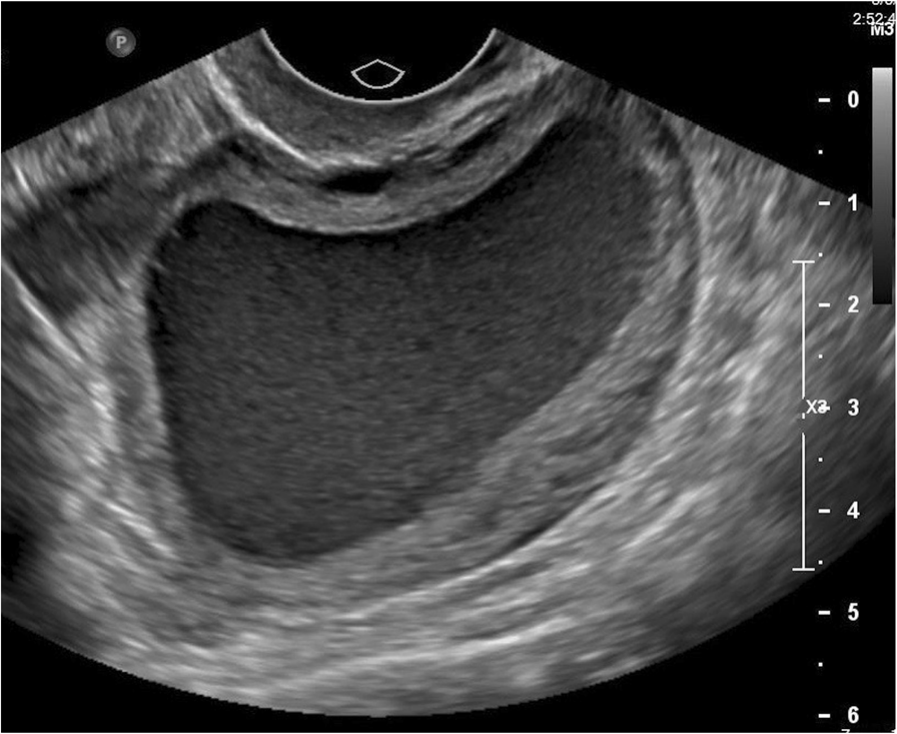
Transvaginal ultrasound. Uterus measuring 7.1×4.6×5.4 cm. The endometrium is distended with homogenous echogenic fluid

**Fig. 2:**
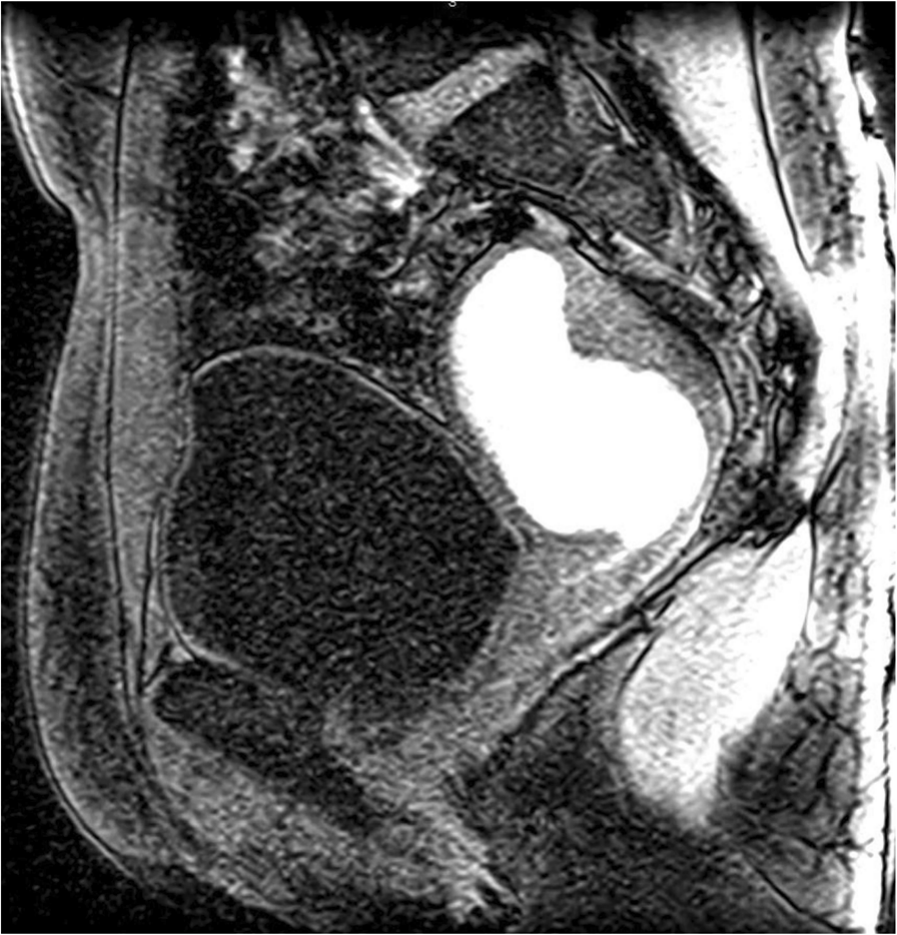
Magnetic resonance imaging. Fluid-filled endometrial cavity and endocervical canal. The canal is non-dilated at 1 to 2 mm

Transabdominal and transvaginal pelvic ultrasound (Fig. 1) was performed and her uterus was measured to be 7.1×4.6×5.4 cm. The endometrium was distended with homogenous echogenic fluid. No discrete mass or congenital anomaly was identified. Her left and right ovaries were of appropriate size with normal echotexture and blood flow bilaterally. She was discharged home with next day Obstetrics and Gynecology (OB/GYN) follow-up and no new findings were reported on subsequent examination. A magnetic resonance imaging (MRI; Fig. 2) was then performed which revealed a fluid-filled endometrial cavity and endocervical canal; however, the canal appeared to be non-dilated at 1 to 2 mm. Hematometra was suspected at this time. Again, there were no discrete obstructing mass or congenital anomaly identified on imaging.

She was scheduled for hysteroscopy followed by fractional dilatation and curettage under general anesthesia. During the procedure, her cervix was sequentially dilated up to 8 mm using a Hegar dilator. Her cervix did not appear to be constricted. A large amount of old bloody material was expelled from her cervix upon dilation and after entrance into her uterine cavity with a hysteroscope. The old bloody material was washed out with sterile saline. Her uterine lining could then be visualized and was noted to be pale and thin, consistent with her history of DMPA use. Both ostia were present and there were no masses or other abnormalities upon visualization. Dilation and curettage by endocervical and endometrial curettings was performed and tissue was sent for pathology. She tolerated the procedures well.

The pathology of her endocervix showed normal benign tissue. The endometrium showed benign inactive to weakly proliferative endometrium and the lower uterine segment had scattered plasma cells consistent with chronic endometritis along with patchy mild nonspecific acute inflammation.

Her symptoms were resolved by the procedure and her 6-month follow-up showed no recurrence of hematometra or other complications. Her DMPA was discontinued at the same time and she switched to oral contraception.

## Discussion

Hematometra presenting as abdominal pain can be a challenging diagnosis. In one case, multiple diagnostic workups including invasive procedures such as exploratory laparoscopy were conducted prior to the appropriate diagnosis [4]. In another case, the rarity of this medical condition resulted in a delay of intervention and contributed to the patient’s prolonged discomfort [2]. Undiagnosed hematometra increases the disease burden of the patient and adds additional financial cost to their medical care [5].

This difficult diagnosis could be further compounded in patients lacking congenital abnormalities and those who have not undergone a prior gynecologic procedure, such as in our patient. In other reports of hematometra, there were additional risk factors to help increase the index of suspicion. One case reported a post-cesarean section hematometra due to uterine adhesion surrounding the internal cervical os [6], and another reported hematometra as a result of leiomyoma obstruction of the uterine outflow tract [5].

To help raise the index of suspicion and aid in the diagnosis of hematometra in an acute abdomen, we recommend the use of abdominal and p﻿elvic ultrasonography. Bedside ultrasound in EDs is becoming ubiquitous in the USA and when combined with sound clinical judgment it can be a useful tool in the evaluation of abdominal pain [7]. Further evaluation of the cause of hematometra can be conducted with MRI. The use of MRI can offer superior contrast resolution that allows for detailed evaluation of the anatomy, without exposing the patient to ionized radiation [8]. Having a detailed knowledge of the patient’s anatomy could aid in the construction of the treatment plan.

There have been a few reports associating the use of DMPA as contraception with cervical stenosis leading to the formation of hematometra [9]. In one study, the use of DMPA resulted in a significantly higher rate of cervical stenosis following LEEP procedure [10]. It is important to note that our patient had used DMPA for 3 years. However, cervical stenosis was not noted based on our observation and the procedure did not require the use of a 2.5 mm Hegar dilator suggesting the absence of cervical stenosis [11]. Although this is based on our anecdotal experience and is a limitation to our case report.

The cause of our patient’s hematometra is unclear given her apparent lack of cervical stenosis, anatomic anomalies, or obstructions. In addition, our patient lacked the known risk factors for chronic endometritis including previous sexually transmitted infections, intrauterine growths, or radiation therapy [12]. This suggests the chronic endometritis noted was most likely caused by the hematometra and not by the cause of her uterine obstruction.

## Conclusions

Although rare, hematometra should be on the differential diagnosis of acute abdominal pain in adolescent females when other common causes have been ruled out. An index of suspicion should be raised when there are positive ultrasound findings and risk factors such as congenital anomalies or prior gynecologic procedures. However, this case highlights that patients may lack risk factors that contribute to hematometra, making the diagnosis potentially challenging. In such instances, complicated diagnostic workups might not always be warranted and a patient’s symptoms could improve immediately following dilation and curettage and the evacuation of old menstrual blood. While not recognized in our patient, it is worthwhile to note the use of DMPA and its possible relation to cervical stenosis as a cause of hematometra.
